# Ankle fractures involving the anterolateral distal tibia: medium-term clinical results of 50 cases

**DOI:** 10.1007/s00068-022-02161-0

**Published:** 2022-11-21

**Authors:** Livia Kroker, Annika Pauline Neumann, Franziska Beyer, Stefan Rammelt

**Affiliations:** Present Address: University Center of Orthopaedics, Trauma and Plastic Surgery, University Hospital Carl Gustav Carus at TU Dresden, Fetscherstrasse 74, 01307 Dresden Germany

**Keywords:** Ankle fracture, Anterior inferior tibiofibular ligament, Anterior malleolus, Syndesmosis

## Abstract

**Purpose:**

The anterolateral distal tibial rim (anterior malleolus, AM) is frequently fractured in malleolar fractures. The aim of this study was to evaluate the medium-term outcomes of malleolar fractures involving the AM.

**Methods:**

Among 100 patients with AM fractures that were treated over a 10-year period, 50 patients were available for follow-up. Outcome was assessed with the Olerud Molander Ankle Score (OMAS), the Foot Function Index (FFI-D), the EuroQol (EQ)-5D-5L Index, the EQ-VAS and the AOFAS Ankle-Hindfoot Score. Type 1 AM fractures (bony syndesmotic avulsions) were fixed surgically with either a suture anchor or a transosseous suture in 11 of 22 cases (50%). Among type 2 AM fractures (with incisura and joint involvement), 68% were treated surgically with screw fixation. All three type 3 AM fractures (anterolateral tibial plafond impaction) were treated surgically with either screw or plate fixation.

**Results:**

At follow-up, the median OMAS was 75, the FFI-D 19, the EQ-5D-5L-Index 0.88, the EQ-VAS 70, and the AOFAS score 93. Assuming that the fracture severity increases from Supination–External Rotation to Pronation–External Rotation and Pronation–Abduction injuries, the AOFAS score (*p* < 0.001), OMAS score (*p* = 0.009), and FFI-D (*p* = 0.041) all showed a significantly inferior clinical outcome with increasing fracture severity. Patients who required surgical revision (*n* = 5) showed a significantly inferior outcome with the OMAS (*p* = 0.019).

**Conclusions:**

A differentiated treatment protocol tailored to dislocation, size, incisura involvement and joint impaction leads to favourable outcomes in complex malleolar fractures involving the AM. More data are needed on the outcome of AM fractures that are still commonly underestimated and overlooked.

## Introduction

Ankle fractures represent almost 10% of all fractures with a reported incidence between 100 and 187 per 100,000 persons per year [1–3]. Epidemiological studies describe an increase in the incidence of ankle fractures in recent years, especially in elderly patients [1–3].

Injuries to the tibiofibular syndesmosis in the wake of ankle fractures may be purely ligamentous in nature or associated with bony avulsions at the distal tibia or fibula [2, 4]. Distally, the trapezoid anterior inferior tibiofibular ligament (AITFL) is an important stabilizer of the syndesmosis. Bony avulsions at the anterior tibial tubercle have been called Tillaux-Chaput fractures and those from the anteromedial fibular tubercle were also called LeFort–Wagstaffe fragments [2, 4–6].

Over the recent years, fractures involving the posterior malleolus (PM) have gained considerable attention [7–9]. It has become apparent that, depending on the individual pathoanatomy, fixation of the posterior tibial fragments recreates the articular surface, facilitates fibular reduction by restoration of the posterior incisura and provides bone-to-bone stabilization of the tibiofibular syndesmosis [2, 6–9]. The same may be assumed about the anterolateral tibial tubercle that has therefore also be termed a fourth [10] or anterior malleolus (AM) [11]. Compared to PM fractures, involvement of the anterolateral tibia is a less frequent condition in adults with a prevalence of 12.6% among all malleolar fractures in a recent study [12]. These fractures are regularly underestimated or overlooked completely in plain radiographs [13–15]. Failure to reduce and fix anterolateral distal tibial fractures reportedly results in malpositioning of the distal fibula in the tibial incisura leading to incongruity of the ankle mortise with possible deleterious consequences [15–18].

**Fig. 1:**
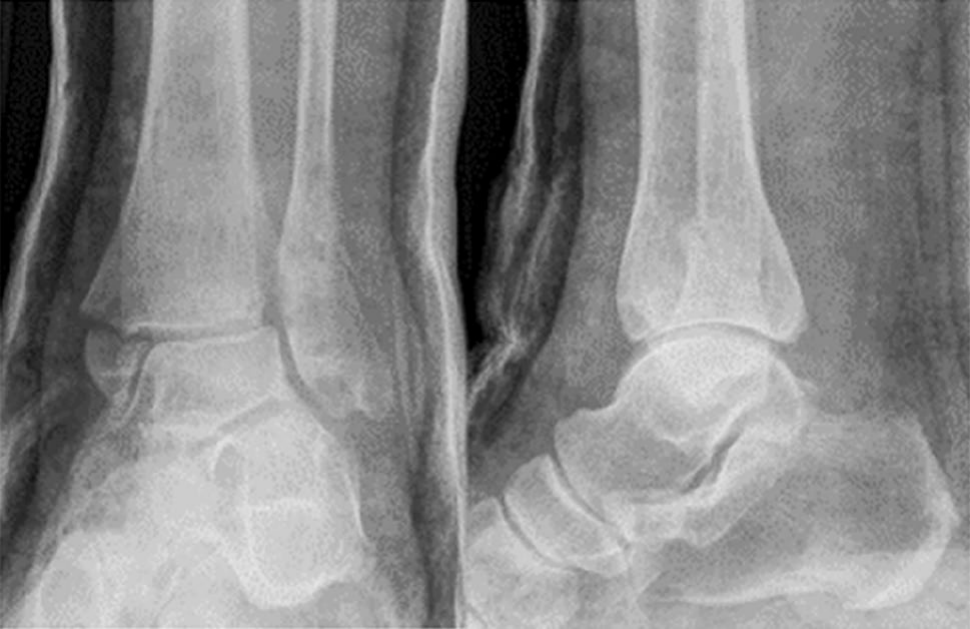
Anteroposterior and lateral radiographs of a 60 year old female patient with a pronation-abduction injury to her left ankle following cast application

In adults, fractures of the anterolateral distal tibia mostly occur as part of more complex ankle fractures with syndesmosis involvement [2, 4, 10, 11]. A substantial portion of these fractures occurs due to a pronation and abduction fracture mechanism (Fig. 1) [4, 12].

While assessment and treatment of Tillaux fractures in adolescents are well described in the literature, there is no general consensus on surgical treatment of fractures and avulsions of the AM in adults. Currently proposed methods include transosseous sutures, suture anchor fixation, screw or plate fixation depending on the size of the fracture and bone quality [11]. Except for case reports and small case series with short-term results, data on outcome are lacking in the literature [16–21]. Therefore, the aim of this study was to evaluate the medium-term outcomes of a sizeable number of malleolar fractures involving the AM in adults and to identify possible prognostic factors.

## Patients and methods

**Fig. 2:**
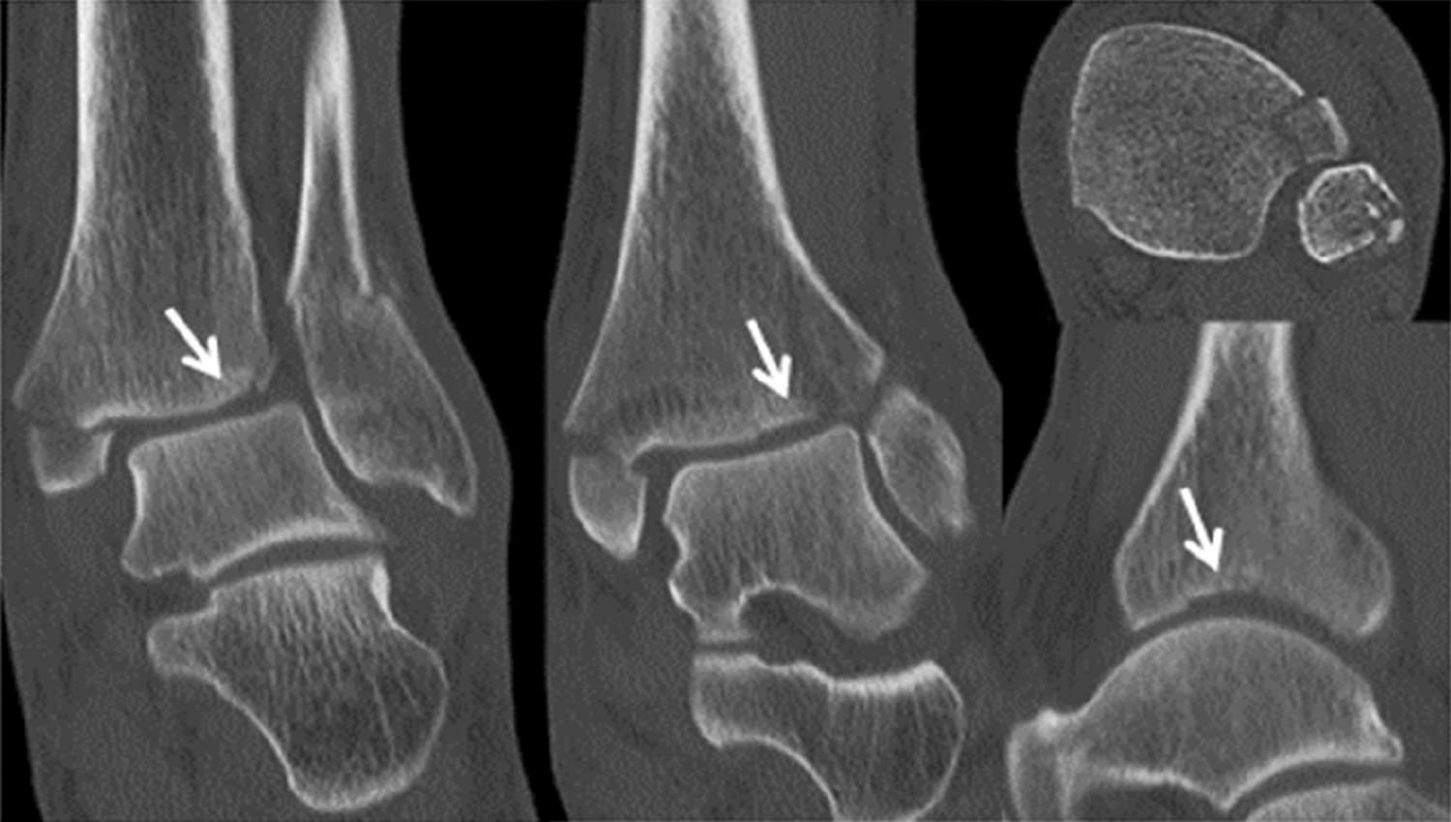
Preoperative CT imaging showing a type 3 anterior malleolar (Chaput) fracture with marginal impaction of the lateral plafond (arrow) as part of a trimalleolar fracture pattern with addition medial and lateral malleolar fracture (same patients in Fig. 1)

A total of 100 patients with surgically treated ankle fractures involving the anterolateral tibial tubercle (anterior malleolus, AM) as diagnosed with CT imaging (Fig. 2) were identified manually from a retrospective review of our institution’s electronic database. All of them were treated between 2008 and 2018 at a Level 1 Trauma Center according to the criteria of the German Trauma Society (DGU) [22, 23].

Exclusion criteria for our clinical study were: age less than 16 years, patients without a complete set of initial radiographs and CT scans, patients with tibial pilon fractures and other accompanying injuries, patients with diabetic neuropathy, patients who were deceased, had moved to a distant or unknown location, and patients who could not appear for a clinical examination for psychosocial, economic or health reasons. This left a total of 50 patients for follow-up (Table 1). The patients were contacted by mail and phone, informed about the study and personally invited to participate. The study protocol was approved by the local institutional review board (ethics committee, reference number EK 340,072,019) and written consent was obtained from all patients.

**Table 1:**
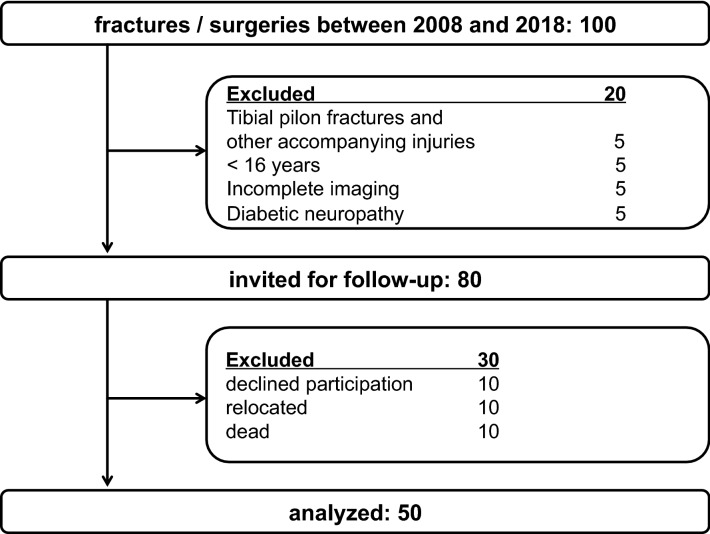
Flowchart for patient inclusion and exclusion

**Fig. 3:**
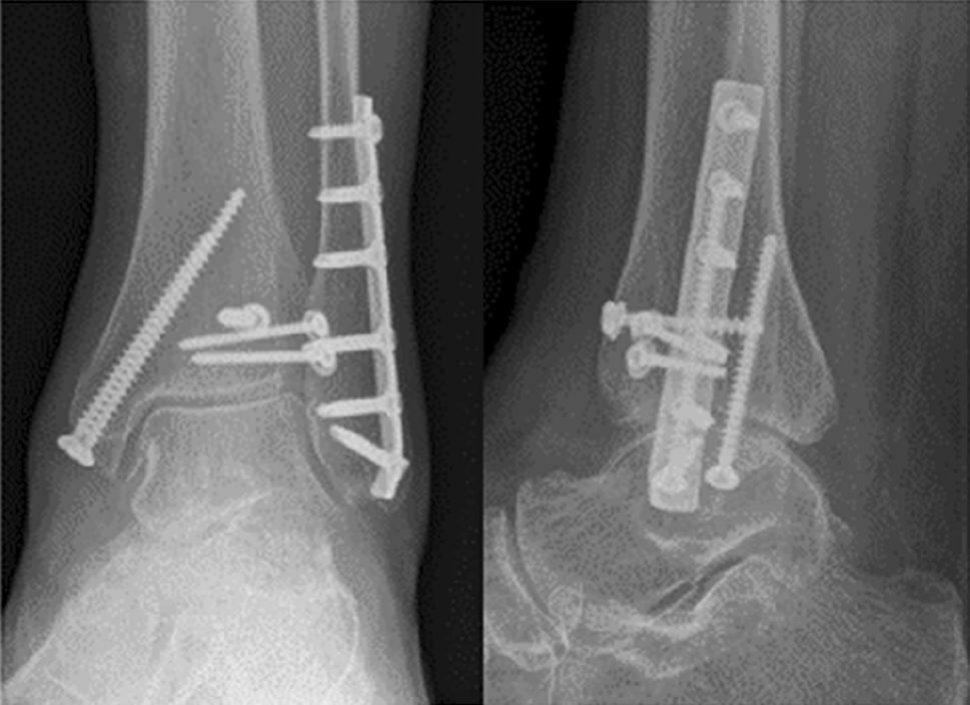
Postoperative anteroposterior and lateral radiographs following open reduction and screw fixation of the anterolateral distal tibia and medial malleolus and plate fixation of the lateral malleolus (same patient in Figs. 1,2)

All fractures were classified according to the Lauge-Hansen system [4]. Fractures of the AM were classified into three types [11]: Type 1 represents an extra-articular avulsion fracture, type 2 an anterolateral tibial fracture involving the joint surface and the fibular incisura, and type 3 an impaction of the anterolateral tibial plafond. Comorbidities and accompanying injuries were recorded. Treatment was tailored to the individual fracture pattern (Fig. 3).

For clinical evaluation, we used the German version of the Foot Function Index (FFI-D) [24], the Olerud Molander Ankle Score [25], the EuroQol (EQ) 5D-5L Index, the EQ-Visual Analogue Scale (VAS) [26] and the American Orthopaedic Foot & Ankle Society (AOFAS) Ankle-Hindfoot Score [27].

Statistical analysis was performed using Microsoft Excel and GraphPad Prism (Microsoft Inc. Seattle, WA, USA). To calculate the EQ-5D-5L Index, we used the Crosswalk Index Value Calculator Software, Version 24OCT2019, from EuroQol [28].

Ordinal data were expressed as medians with interquartile range (IQR). We used the Mann–Whitney test to measure the central tendencies of two groups e.g. to determine if revision surgery or comorbidities (diabetes, osteoporosis) result in worse clinical outcomes. We used the Spearman rank correlation test for correlation between ranked ordinal variables such as the fracture severity and clinical outcome score results. A *p*-value lower than 0.05 was considered statistically significant. We did not correct for multiple testing as we conducted an exploratory study to identify factors that potentially influence clinical outcome. The reported *p*-values should therefore be seen as indicative.

## Results

Of the 50 patients that met the inclusion criteria and were available for clinical evaluation, the average follow-up was 33.5 months (range 11 to 131 months). 19 patients had an ankle fracture on the right side and 31 on the left side. The mean age at the time of surgery was 74.6 years (Median 65, IQR 21.5). There was no correlation between the type of the AM fracture and patient age (*p* = 0.79, rho =  – 0.0383). Of all patients, 54% (27) were female and 46% (23) were male.

Twenty-nine patients suffered a twisting injury to the ankle, 13 patients suffered a fall from a height, and 8 patients sustained a traffic accident. The mechanism of injury had no significant influence on the clinical outcome with any of the scores.

Overall, a type 1 AM fracture was seen in 22 patients, type 2 in 25 and type 3 in 3 patients. Patient details and patterns of injury for all three types are provided in Table 2. At final follow-up, the median FFI-D was 19 (IQR 39.4). The median OMAS was 75 (IQR 46.2). The median EQ-5D-5L Index was 0.878 (IQR 0.212). The median EQ-VAS was 70 (IQR 33.8). The median AOFAS was 93 (IQR 20.8). With the numbers available, there was no significant correlation between the type of the anterolateral tibia fracture (AM) and the result with any of the scores.

**Table 2:** Injury characteristics for the 3 different types of observed anterior malleolar (AM) fractures

Pathoanatomy of the AM fracture	Type 1 (avulsion of AITFL)	Type 2 (involvement of incisura and joint surface)	Type 3 (anterolateral plafond impaction)
Total number	22	25	3
Female	11	15	1
Male	11	10	2
Right	7	12	0
Left	15	13	3
Osteoporosis	5	5	0
Diabetes	5	5	0
Associated lateral malleolar fracture	20	24	3
Associated medial malleolar fracture	19	18	2
Associated posterior malleolar fracture	20	17	2
SER fracture	8	8	0
PER fracture	7	9	2
PA fracture	5	8	1

*SER* supination – external rotation, *PER* pronation – external rotation, *PA* pronation – abduction

An associated fracture of the lateral malleolus was present in 47 of 50 patients (94%), an associated fracture of the medial malleolus in 39 (78%), a rupture of the deltoid ligament in one (2%), an associated fracture of the posterior malleolus (PM) in 39 (78%). A quadrimalleolar fracture pattern [29], that is, a fracture of the medial, lateral, posterior and anterior malleolus, was seen in 31 of 50 patients (62%). A trimalleolar fracture pattern was seen in 11 patients (22%). The median values of the OMAS (70 vs. 75), FFI-D (20.98 vs. 38.10) and the AOFAS (85 vs. 85) score showed no tendency towards inferior clinical outcome in quadrimalleolar fractures compared to trimalleolar fractures (*p* > 0.05 for all scores).

According to Lauge-Hansen, 16 patients sustained a supination-external rotation (SER) fracture, 18 patients sustained a pronation-external rotation (PER) fracture, and 14 patients sustained a pronation-abduction (PA) fracture. In two patients, the fracture pattern could not be classified according to the Lauge-Hansen system because they sustained a fracture of the AM combined with a fracture of the PM. If one assumes that the fracture severity increases from SER to PER and PA injuries, then the AOFAS score (*p* < 0.001, rho =  – 0.505), OMAS score (*p* = 0.009, rho =  – 0.369), and FFI-D (*p* = 0.041, rho = 0.296) all showed a significantly inferior clinical outcome with increasing fracture severity. The EQ-5D-5L Index (*p* = 0.05, rho =  – 0.333) and EQ-VAS (*p* > 0.05, rho =  – 0.244) showed a trend towards the same direction.

22 patients with fracture-dislocations were initially treated with closed reduction and external fixation for temporary stabilization and soft tissue consolidation. A single shot antibiotic was administered for all procedures. 23 patients received a peripheral nerve block before surgery. A lateral approach was used in 38 cases and an anterolateral approach was used in 8 cases to expose the AM. Overall, forty-five patients received locking plates, and 5 patients received screw fixation. None of these factors had a significant influence on any of the scores.

**Fig. 4:**
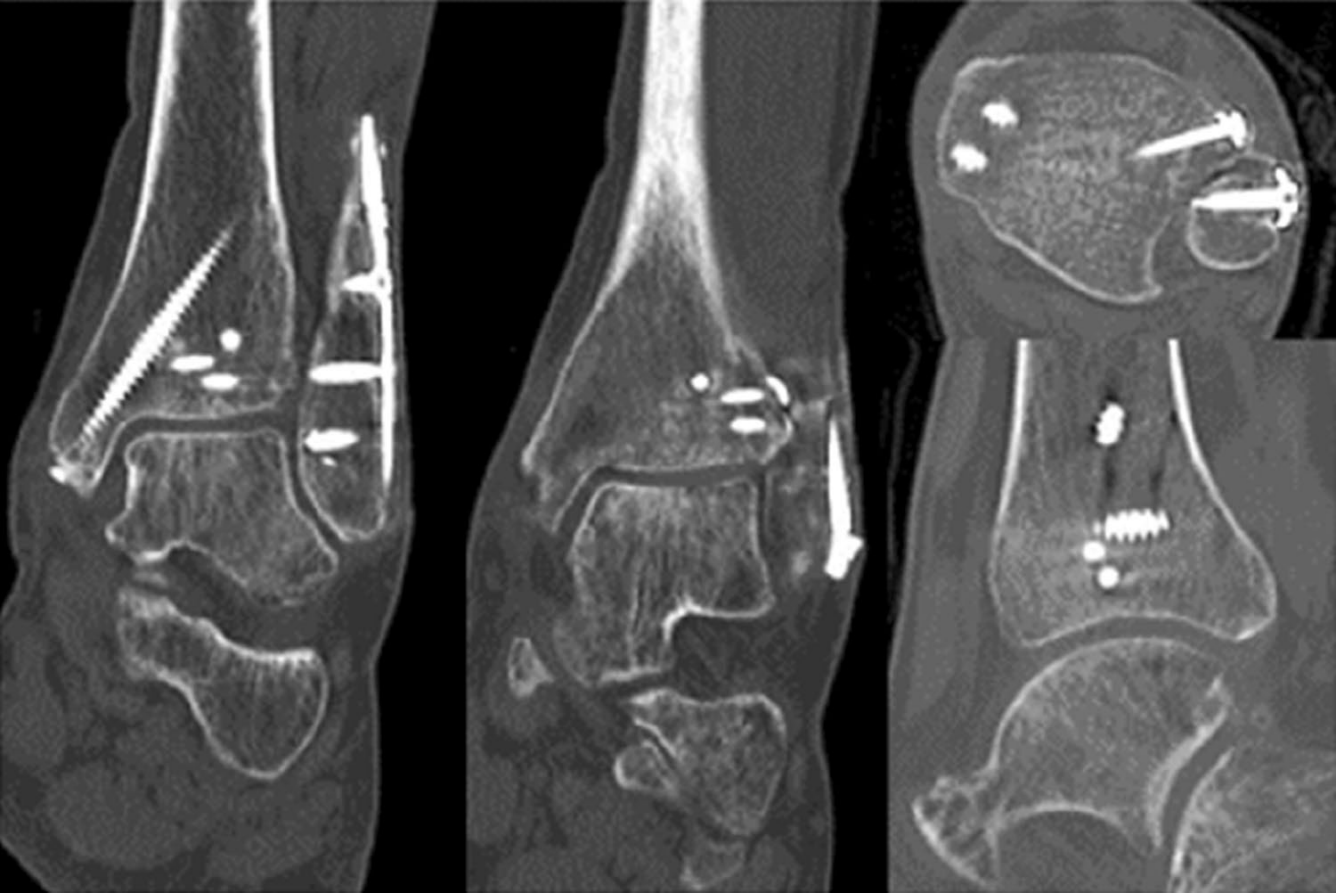
Postoperative CT imaging showing anatomic reduction with restoration of the incisura, the lateral plafond and ankle mortise (same patient in Figs. 1,2, 3)

Type 1 AM fractures were surgically fixed with either a suture anchor or a transosseous suture in 11 of cases (50%). The majority (68%) of type 2 AM fractures were surgically treated with screw fixation. All type 3 AM fractures were treated surgically with either screws or a plate (Fig. 4). Treatment according to AM fracture type is detailed in Table 3. The results of the outcome scores are summarized in Table 4.

**Table 3:** Mode of fixation according to the type of AM fracture

AM fracture	Type 1 (*n* = 22)	Type 2 (*n* = 25)	Type 3 (*n* = 3)
No fixation	5	2	–
Transosseous suture	2	4	–
Suture anchor	9	2	–
Screw	3	17	2
Plate	–	–	1
Additional syndesmotic screw	3	5	1

**Table 4:** Median (IQR) outcome scores according to the type of AM fracture

AM fracture	type 1 (*n* = 22)	type 2 (*n* = 25)	type 3 (*n* = 3)	*p*	rho
OMAS	82.50 (40)	75 (45)	55 (65)	0.192	– 0.188
FFI-D	13.58 (34.17)	31.48 (49.5)	17.28 (34.57)	0.339	0.138
EQ-5D-5L	0.914 (0.212)	0.845 (0.245)	0.910 (0.19)	0.394	– 0.147
EQ-VAS	90 (35)	70 (25)	80 (30)	0.958	0.009
AOFAS	96.50 (18)	85 (37.5)	93 (14)	0.381	– 0.127

In nine patients, a syndesmotic screw fixation was performed in addition to malleolar fracture fixation. This did not result in a different clinical outcome (*p* > 0.05 in all 5 clinical outcome test categories). In 7 patients, a non-displaced or minimally-displaced (< 2 mm) AM fracture was left unfixed. Of the seven unfixed AM fractures, five were classified as type 1. In four of these, the syndesmosis was stressed and the ankle mortise evaluated as stable intraoperatively following surgical PM fixation. In the remaining unfixed type 1 fracture, the syndesmosis was visualized intraoperatively and evaluated as stable. Two untreated AM fractures were classified as type 2. One of them was an open fracture with poor local soft tissue conditions. In the other patient, it was decided not to attempt fixation of the AM because of severe osteoporosis in a 91-year-old female patient. Final outcome of the unfixed AM fractures was not significantly different from those with AM fracture fixation.

With respect to relevant comorbidities, 10 patients (20%) suffered from osteoporosis and 10 patients (20%) suffered from diabetes. Two patients were smokers. 17 patients suffered from arterial hypertension, 3 of them additionally from coronary artery disease and one of them additionally from heart failure. The presence of comorbidities did not result in a significantly worse outcome with any of the scores.

In terms of complications, two patients reported diminished sensation in the scar area, two patients had persistent oedema at the ankle, one patient suffered from a pin infection following external fixation that could be treated conservatively, and 5 patients required surgical revision due to malpositioning of the ankle (*n* = 4) and wound healing problem (*n* = 1). One patient with a PER injury showed a malposition of the fibula (shortening, external rotation). Surgical revision with reduction of the fibula, fixation with syndesmotic screws and transosseous suture of the anterior syndesmosis was performed. Another patient with a PER injury showed a malposition of the medial malleolus and the fibula. The malposition of the medial malleolus was corrected, the length of the fibula was corrected, and the tubercule de Chaput was fixed with a screw. Another patient with a PA injury and dislocated tubercule de Chaput and malalignment of the fibula (shortening, malrotation) also received revision of the fibula and screw fixation of the tubercule de Chaput. Another patient with PA injury with impacted articular surface of the anterolateral tibia received surgical revision. The articular surface was elevated and fixation was performed using a plate. An 80-year-old female patient with a PA 3 injury and secondary diagnoses of diabetes and osteoporosis showed impaired wound healing. Postoperative immobilization in a cast was performed, which was removed early due to discomfort. A 2 × 1 cm skin defect was surgically debrided and treated with sutures. The overall rate of wound healing disorders or infections was 4%. The surgically revised ankle fractures showed a significantly inferior outcome with the OMAS (*p* = 0.019). Potential prognostic factors and respective outcome scores are summarised in Table 5.

**Table 5:** Correlation of potential prognostic factors and outcome scores: *p*-values and medians (IQR) are provided, significant values are printed in bold

Score	OMAS	FFI-D	EQ-5D-5L	EQ-VAS	AOFAS
Surgical AM fixation (n = 43) vs	75 (45)	19 (38.56)	0.91 (0.212)	75 (39)	93 (20)
no AM fixation (*n* = 7)	80 (40)	14.19 (72.84)	0.828 (0.245)	80 (50)	85 (32)
*p*-value	*p* = 0.829	*p* = 0.798	*p* = 0.667	*p* = 0.939	*p* = 0.457
No additional syndesmotic screw (*n* = 34) vs	75 (45)	19.99 (38.22)	0.91 (0.212)	77.5 (31.25)	93 (21.75)
Additional syndesmotic screw (*n* = 9)	100 (40)	0 (45.55)	1.0 (0.192)	70 (35)	100 (17)
*p*-value	*p* = 0.288	*p* = 0.583	*p* = 0.480	*p* = 0.296	*p* = 0.287
No diabetes (*n* = 40) vs	77.5 (45)	19 (41.14)	0.91 (0.208)	80 (37.5)	93 (19.5)
Diabetes (*n* = 10)	72.5 (38.75)	18 (35.86)	0.817 (0.195)	55 (35)	89 (40)
*p*-value	*p* = 0.633	*p* = 0.735	*p* = 0.234	*p* = 0.127	*p* = 0.465
No osteoporosis (*n* = 40) vs	77.5 (50, 60–95)	19.99 (42.08)	0.91 (0.2075)	80 (39.7)	94 (22.25)
Osteoporosis (*n* = 10)	72.5 (25)	14.5 (37.76)	0.817 (0.225)	65 (42.5)	89 (27.25)
*p*-value	*p* = 0.874	*p* = 0.666	*p* = 0.149	*p* = 0.618	*p* = 0.583
No revision surgery (*n* = 45) vs	75 (45)	19 (38.33)	0.91 (0.212)	80 (39.5)	93 (20)
Revision surgery (*n* = 5)	40 (77.5)	18 (41.98)	0.845 (0.307)	75 (55)	93 (44.5)
*p*-value	***p* = 0.019**	*p* = 0.348	*p* = 0.072	*p* = 0.115	*p* = 0.132
Work-related accident (*n* = 44) vs	77.5 (45)	17.64 (37.99)	0.91 (0.208)	77.5 (39.75)	94 (18)
Non work-related accident (*n* = 6)	60 (26.25)	39.26 (24.65)	0.792 (0.34)	70 (27.5)	80 (31.75)
*p*-value	*p* = 0.190	*p* = 0.112	*p* = 0.354	*p* = 0.732	*p* = 0.094
Trimalleolar (*n* = 11) vs	75 (45)	38.1 (35)	0.828 (0.104)	60 (20)	85 (27)
Quadrimalleolar (*n* = 31) fractures	70 (45)	20.98 (42)	0.91 (0.245)	80 (50)	85 (27)
*p*-value	*p* = 0.665	*p* = 0.319	*p* = 0.473	*p* = 0.333	*p* = 0.789

## Discussion

Ankle fractures with involvement of the anterolateral tibia (Tubercule de Tillaux-Chaput, anterior malleolus, AM) in adults are predominately part of complex ankle fractures [11–13, 29–31]. They represent syndesmotic avulsion, possible incisura and joint involvement, and even tibial plafond impaction [11]. Consequently, trimalleolar fractures with additional involvement of the AM have been termed quadrimalleolar fractures and those involving the anterior fibular rim “quadrimalleolar equivalent” [12, 31].

Despite early and exhaustive studies on that subject [4, 30], there is a lack of current clinical data. To our best knowledge, we report the largest patient group so far with ankle fractures involving the anterolateral distal tibia in adults.

Pronation injuries according to Lauge-Hansen are particularly unstable injuries often resulting in irregular fracture patterns [4]. In a recent pathoanatomic analysis of 1379 malleolar fractures, AM fractures were significantly more frequent in pronation injuries [12]. In a radiographic analysis of 140 cases, type 3 AM fractures with anterolateral plafond impaction were significantly associated with PA fractures while type 1 avulsion injuries were more frequent in supination injuries [12]. Our clinical study shows that with increasing fracture severity according to Lauge-Hansen, clinical outcome of ankle fractures with AM involvement becomes worse, if PA fractures are considered the most severe ones.

Fractures of the anterolateral distal tibia are among the most frequently overlooked features in malleolar fractures and, if displaced, a cause for revision surgery to correct tibiofibular malalignment [13–18, 32]. The three-dimensional fracture pattern remains elusive even in adequate radiographic projections, in particular, the involvement of the fibular incisura, intercalary fragments and displacement or impaction of the articular surface [12]. Therefore, in analogy to PM involvement, the use of CT scanning is recommended, in complex ankle fractures if an AM fracture is suspected [11, 15].

A multitude of studies has shown that correct placement of the distal fibula within the tibial incisura is of utmost relevance for outcome in malleolar fractures with an unstable syndesmosis [2, 33–36]. Consequently, much attention has been paid to PM fractures because it became apparent, that anatomic reduction and direct, stable fixation even of smaller fragments restores syndesmotic stability and facilitates fibular reduction through restoration of the integrity of the incisura [6–9, 36]. For the same reasons, it seems logical to perform open reduction and direct fixation of displaced anterior bony syndesmosis avulsions. Depending on shape, size, dislocation, involvement of the incisura and impaction, we performed transosseous suture or anchor fixation for displaced type 1 AM fractures, screw fixation for displaced type 2 AM fractures, and elevation of the depressed joint surface followed by screw or small buttress plate fixation for all type 3 AM fractures [11]. Alternatively, for isolated Tillaux-Chaput fractures, arthroscopically-assisted surgery has been recommended [11, 20, 37]. When performing open reduction and internal fixation for lateral malleolar fractures, we have a low threshold for exploring the anterior syndesmosis to detect and anatomically fix avulsions from either the distal tibia or fibula. Closed reduction of syndesmotic disruptions and percutaneous screw fixation, for instance in Maisonneuve injuries, is discouraged because it carries a significant risk of malreduction [38]. An unrecognized, interposed AM fragment may even render closed reduction impossible [31].

Excellent results have been reported following open or arthroscopically-assisted fixation of isolated Chaput fractures [20, 37]. However, because of the relative paucity of these particular injuries in adults, experience is limited to case reports and small case series. Zhao et al. report good to excellent short-term results in 12 of 15 patients with malleolar fractures including the AM [40]. Birnie et al. [16] found avulsions from the anterior tibial tubercle in 25.8% of 252 operatively treated Wagstaffe fractures. More than half of the AM fractures had a fragment size of more than 5 mm and were fixed directly. Revision surgery was needed in 6.2% of cases, exclusively for unfixed fragments [16]. Zhang and colleagues [5] found chondral lesions at the lateral talar dome in 8 of 13 patients with concomitant anterior tibial and fibular avulsion fractures. The results were good at 14 months with an average OMAS of 82. In a recent paper, Wei et al. [39] describe 15 surgically treated patients with AM fractures who were followed up for an average of 38.1 months. The average age was 45.6 years. The AOFAS score, that is not validated, was 85.6. The results of our clinical study are good to moderate which may reflect the longer follow-up and complex fracture pattern with 22% trimalleolar and 62% quadrimalleolar fractures. The scores of both groups were not statistically significant which is in concordance with a recent clinical study on long-term results of 100 ankle fractures with PM involvement [36]. When compared to the results of this study, the scores for malleolar fractures involving the AM are slightly inferior to those involving the PM. One possible reason might be the higher patient age in patients with AM fractures that has been confirmed in a large patient cohort [12].

**Fig. 5:**
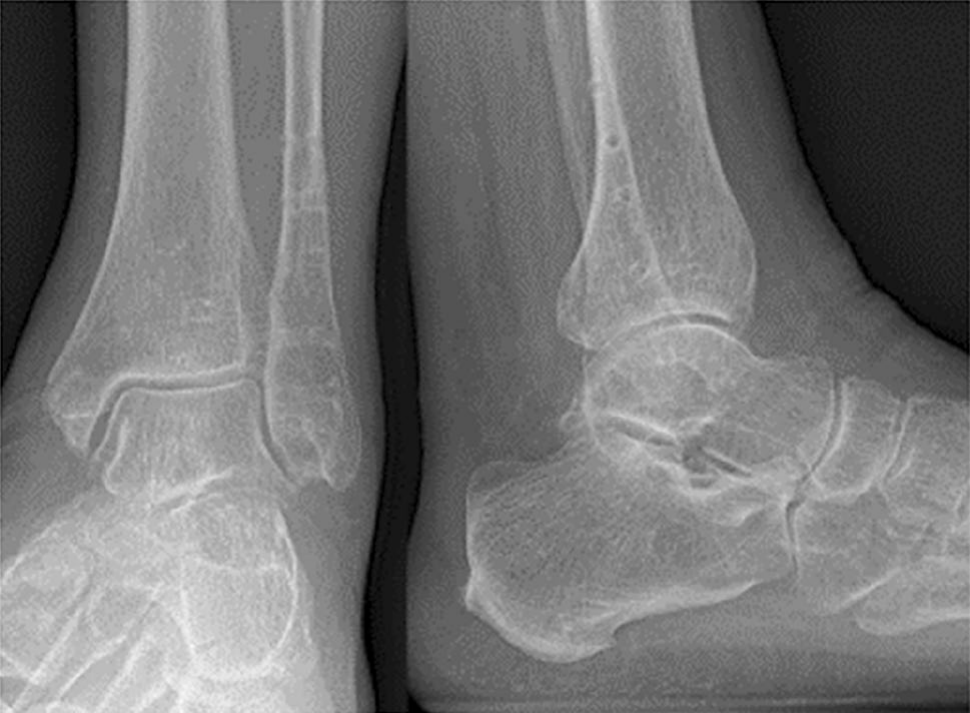
Standing anteroposterior and lateral radiographs at a follow-up of 19 months after the injury and implant removal showing a congruent ankle joint without radiographics signs of posttraumatic arthritis (same patients in Figs. 1,2,3,4)

In contrast to PM fractures, principles for fixation of displaced AM fractures have only been proposed recently. There are several reports on malunited or malpositioned Chaput fragments requiring revision [6, 15–18, 32]. In the present study, patients requiring surgical revision (mostly for initial malreduction) had significantly inferior scores. From the available data, it seems, that a treatment algorithm tailored to the individual fracture pattern and displacement provides good outcomes even for complex fracture patterns (Fig. 5). While it seems logical, to award the AM a similar attention as the PM with respect to both recognition, assessment and treatment, larger comparative clinical studies with long-term follow-up are needed to confirm these statements.

Study limitations include the retrospective study design, loss to follow-up and the relatively low number of patients per type of AM fracture and treatment group with potential introduction of alpha error into the statistical analysis. Because we treated all displaced AM fractures operatively, we cannot tell if this treatment is superior to other treatment options with our data**.** Still, our study represents by far the largest series and longest follow-up for patients with ankle fractures involving the anterolateral distal tibia.

In conclusion, favourable outcomes may be achieved in complex ankle fractures involving the AM with a treatment algorithm based on the individual on dislocation, shape, size, impaction, and incisura involvement of the of the AM fractures that seem to be fraught with a slightly inferior prognosis than PM fractures. The proposed CT-based classification [11] that has been validated with a high intra- and interobserver reproducibility [12] may be used to guide treatment. More data is needed on the outcome of AM fractures that are still commonly underestimated and overlooked in the setting of malleolar fractures in adults.

## Data Availability

The datasets generated and analysed during the current study are available from the corresponding author on reasonable request.
